# Development and validation of a predictive score for ICU delirium in critically ill patients

**DOI:** 10.1186/s12871-021-01259-z

**Published:** 2021-02-05

**Authors:** Huijuan Zhang, Jing Yuan, Qun Chen, Yingya Cao, Zhen Wang, Weihua Lu, Juan Bao

**Affiliations:** 1 Department of Intensive Care Unit, Yijishan Hospital, First Affiliated Hospital of Wannan Medical College, Wuhu, 241001 Anhui China; 2 Department of Nursing, Yijishan Hospital, First Affiliated Hospital of Wannan Medical College, Wuhu, 241001 Anhui China

**Keywords:** Intensive care unit, Delirium, Incidence, Risk factors, Prediction

## Abstract

**Background:**

The incidence of delirium in intensive care unit (ICU) patients is high and associated with a poor prognosis. We validated the risk factors of delirium to identify relevant early and predictive clinical indicators and developed an optimized model.

**Methods:**

In the derivation cohort, 223 patients were assigned to two groups (with or without delirium) based on the CAM-ICU results. Multivariate logistic regression analysis was conducted to identify independent risk predictors, and the accuracy of the predictors was then validated in a prospective cohort of 81 patients.

**Results:**

A total of 304 patients were included: 223 in the derivation group and 81 in the validation group, 64(21.1%)developed delirium. The model consisted of six predictors assessed at ICU admission: history of hypertension (RR = 4.367; *P* = 0.020), hypoxaemia (RR = 3.382; *P* = 0.018), use of benzodiazepines (RR = 5.503; *P* = 0.013), deep sedation (RR = 3.339; *P* = 0.048), sepsis (RR = 3.480; P = 0.018) and mechanical ventilation (RR = 3.547; *P* = 0.037). The mathematical model predicted ICU delirium with an accuracy of 0.862 (*P* < 0.001) in the derivation cohort and 0.739 (P < 0.001) in the validation cohort. No significant difference was found between the predicted and observed cases of ICU delirium in the validation cohort (*P* > 0.05).

**Conclusions:**

Patients’ risk of delirium can be predicted at admission using the early prediction score, allowing the implementation of early preventive interventions aimed to reduce the incidence and severity of ICU delirium.

## Introduction

Delirium is a disturbance of consciousness characterized by an acute onset and a fluctuating course of impaired cognitive functioning. ICU delirium is a vital issue as its incidence rang from about 20 to 80% and highly depends on the population studied and the diagnostic method used [1, 2]. Some studies showed that the incidence of postoperative delirium is approximately 31–50% [3]; furthermore, the higher incidence rate is found in the elderly patients and ICU patients undergoing mechanical ventilation [4]. Delirium are prone to prolonged hospital stay and mechanical ventilation, increased amount of sedative drugs and costs [2, 5].

The delirium risk factors reported by the 2018 PADIS (Pain Agitation Delirium Immobility Sleep) guidelines include pre-existing dementia, history of hypertension or alcoholism, serious illness at admission, coma, and benzodiazepine use [6]. Bart Van Rompaey et al. [7] conducted a multicentre study involving 523 patients, found that neither age, age over 65 years, nor gender showed a relation to the onset of delirium. In addition, a history of drinking with more than three units per day was related to the development of delirium in ICU patients, which was in line with the finding by Spies et al. [8] that drinking predisposed individuals to perceptual disturbances. Although delirium monitoring is advocated in numerous evidence-based guidelines as part of routine clinical care, it is still not widely and consistently performed at the bedside in different patient care settings. Currently, several scoring systems have been proposed, such as the PRE-DELIRIC model, which was developed in 2012 by van den Boogaard et al. [9] and based on 10 readily available risk factors. However, the usefulness of the model is limited by the fact that it requires predictors obtained within 24 h of ICU admission. Another study [10] showed that patients who suffered from nervous system disease had a higher incidence of delirium by predicting delirium in critically ill patients using 11 related factors, and determined that prophylaxis with Dexmedetomidine Hydrochloride in delirium ICU patients was beneficial. Moreover, the PREDICt model is designed to detect POD in surgical ICU patients, but it’s difficult to exactly available for the general intensive care patients [11]. Correlational research [12] suggested that screening could increase the rate of its diagnosis to 64%, and the early essential intervention could reduce both the incidence of delirium and its duration and complications. This highlights the importance of evaluating the performance of such models which based on identified risk factors for delirium in ICU patients.

Accordingly, the aim of this study was to develop and validate a simple delirium predictive model. We further evaluate the predictive value of the model regarding the development of delirium.

## Method

### Design and sample

Prospective observational single study carried out in mixed intensive care units for adult patients. The study received institutional approval by Research Ethical Committees, and informed consent of the patients’ families was required. All consecutive patients who were aged ≥18 years, and admitted to stay in ICU for more than one day between September 2015 and September 2016 were included in the study. The assessment of mental illness was based on the American Psychiatric Diagnostic and Classification Manual, Fourth Edition, and cranial CT examination was used to determine organic encephalopathy. The exclusion criteria were [1] neurological disease or organic encephalopathy [2]; a history of dementia, depression or schizophrenia [3]; a history of preoperative cognitive impairment [4]; a sustained coma during complete ICU stay [5]; death at the time of screening.

### Data collection

Possible risk factors related delirium avaliable after patients were enrolled in the study were: age, APACHE-II scores (Acute physiology and chronic health evaluation) in the 24 h after ICU admission, mechanical ventilation, metabolic acidosis, history of hypertension, history of diabetes, history of heart disease, history of peptic ulcer toxaemia, history of pulmonary dysfunction, the application of Benzodiazepines, alcohol abuse, nicotine, acidosis, the types of surgery and the duration, arterial blood gas analysis after the outcome -delirium, sepsis, hypoxaemia (see Table 1 data supplement for exact definitions).

**Table 1:** Characteristics of derivation cohort

Variable	Delirium (*n* = 46)	No delirium (*n* = 177)
Age > 65 years, [n(%)]	22(47.8)	61(34.5)
Male/Female, [n(%)]	35/11	110/67
History of hypertension, [n(%)]	24(52.2)	48(27.1)
Heart disease, [n(%)]	10(21.7)	31(17.5)
History of pulmonary dysfunction, [n(%)]	5(10.9)	18(10.2)
Alcohol abuse, [n(%)]	7(15.2)	15(8.5)
History of nicotine, [n(%)]	12(26.1)	29(16.4)
History of peptic ulcer, [n(%)]	2(4.3)	1(0.6)
Hypoxaemia, [n(%)]	28(60.9)	37(20.9)
Hypotension, [n(%)]	27(58.7)	65(36.7)
Deep sedation, [n(%)]	33(71.7)	57(32.2)
Benzodiazepines, [n(%)]	30(65.2)	42(23.7)
Mechanical ventilation, [n(%)]	41(89.1)	74(41.8)
Metabolic acidosis, [n(%)]	17(37.0)	39(22.0)
Sepsis, [n(%)]	29(63)	52(29.4)
Surgery, [n(%)]	23(50)	70(39.5)

(History of pulmonary dysfunction: chronic obstructive pulmonary disease or bronchial asthema before ICU admission; Alcohol abuse: more than 150 ml average daily for ten years; History of nicotine: more than 20 cogaretters average daily for ten years; Hypoxaemia: PaO_2_ < 60 mmHg or SpO2 < 90% and successive oxygen therapy or mechanical ventlation more than 24 h after ICU admission; Hypotension:MAP < 65 mmHg and need vascoactive drug support; Deep sedation**:** a Richmond agitation sedation score (RASS) value of −3 to −5 and using sedatives continuously for more than 3 days after study enrollment**;** Benzodiazepines: application of Benzodiazepines for 24 h after ICU admission; Metabolic acidosis: PH < 7.35 or Lac> 2.2 mmol/l; Sepsis: met the 2015 sepsis definition of Systemic Inflammatory Response Syndrome)

**Table 2:** Independent predictors of delirium in ICU derived from univariate regression analysis performed on the derivation cohort (Count data)

Risk factor	Delirium group (n = 46)	No delirium group (*n* = 177)	χ2	P
Male, [n(%)]	35(76.1)	110(62.1)	3.120	0.077
History of hypertension, [n (%)]	24(52.2)	48(27.1)	10.484	0.001
Pulmonary dysfunction, [n(%)]	5(10.8)	18(10.2)	0.019	0.889
Hearing impairment, [n(%)]	4(8.7)	17(9.6)	–	1.000
History of diabetes, [n(%)]	5(10.9)	20(11.3)	4.746	0.029
Hypoxaemia,[n(%)]	28(60.9)	37(20.9)	28.238	0.000
Hypotension,[n(%)]	27(58.7)	65(36.7)	7.273	0.007
Deep sedation,[n(%)]	33(71.7)	57(32.2)	23.709	0.000
benzodiazepines, [n(%)]	30(65.2)	42(23.7)	28.746	0.000
Metabolic acidosis, [n(%)]	17(37.0)	39(22.0)	4.323	0.038
History of peptic ulcer, [n(%)]	2(4.3)	1(0.6)	–	0.109
Sepsis, [n(%)]	29(63)	52(29.4)	17.890	0.000
Surgery, [n(%)]	23(50)	70(39.5)	1.641	0.200
CPOT≤3 [n(%)]	40(91.3)	162(93.8)	0.893	0.345

(CPOT:Criticalcare Pain Observation Tool)

### Delirium assessment

Several tools are available to assess delirium in intensive care patients, of which the CAM-ICU has the highest sensitivity and specificity [13], the inter-rate reliability of the delirium screenings by the intensive care nurses was 0.92–0.96 [14]. All consecutive patients were screened by well-trained ICU nurses twice a day (at 9:00 AM-11:00 AM and 03:00 PM-05:00 PM) using the CAM-ICU. We determined a priori that an inter-rater reliability of 0.85 Cohen’s kappa indicated reliable data. Each patients were assessed for delirium lasting five days or until discharge from ICU. We defined patients as having delirium when they had at least one positive CAM-ICU screening during their intensive care stay.

### Sample size

With an anticipated delirium incidence of 20 to 40% and an attrition rate of 10%, we aimed to enroll 1.962*0.4*0.6/(0.15*0.4)^2^ /0.9 = 286 patients. The first aim was to develop the model, and the second was to validate the model.

### Statistical analysis

Cohen’s Kappa test was used to detect the inter-rater reliability of delirium screening by trained intensive care nurses. Measurement data were tested for a normal distribution using the homogeneity of variance test and Kolmogorov-Smirnov Test. Continuous variables with a normal distribution were presented as the mean ± standard deviation (SD). Analysis was carried out using Student’s t-test. Categorical variables were recorded, percentages were calculated, and the χ2 test was used for these analyses. Fisher’s exact was used to test for T < 1 or *n* < 40. Each score’s discriminatory power was assessed by calculating the area under the receiver operating characteristic (ROC) curve (AUROC). The Hosmer-Lemeshow test was used to determine whether there were differences between the predicted and observed cases of ICU delirium in the validation cohort. Comparisons in which *P* < 0.05 were considered statistically significant.

## Results

### Development of prediction model

**Fig. 1:**
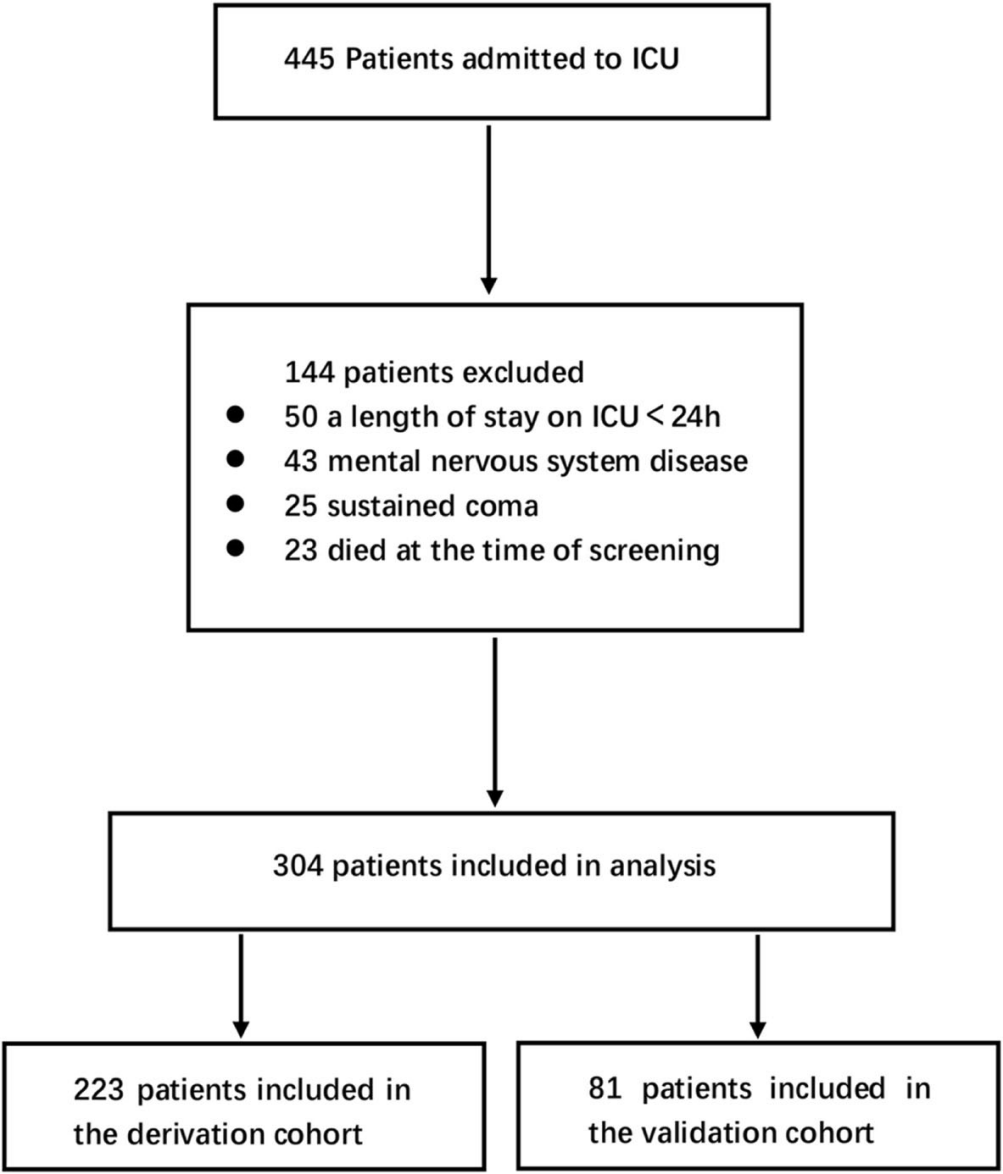
Patient inclusions and exclusions. The number of patients excluded because of each exclusion criterion is shown

A total of 445 consecutive patients were screened,141 of whom were excluded (Fig. 1). The most frequent reasons for exclusion were followed by a length of stay in the ICU < 24 h 50, mental nervous system disease 43, sustained coma 25, and died at time of screening 23. The cohort consisted of 304 patients, 223 of whom were allocated to the derivation cohort (September 2015 to January 2016) while the remaining 81 patients were allocated to the validation cohort (February 2016 to April 2016). We recorded each patients’s data in forms (Table 1-3), and we used data from first 223 critically ill patients to construct the predictive model, which consist of 6 risk factors (Table 4).

**Table 3:** Independent predictors of delirium in ICU derived from univariate regression analysis performed on the derivation cohort (Measurement data)

Risk factor	Delirium group (n = 46)	No delirium group (n = 177)	t	P
Age (yr, ®x ± SD)	62.80 ± 17.31	57.63 ± 13.88	−2.134	0.034
WBC (10^9^/L)	14.89 ± 8.11	11.38 ± 4.81	2.807	0.005
PLT (10^9^/L)	116.41 ± 64.19	149.03 ± 90.31	2.301	0.022
CRP (mmol/L)	173.97 ± 167.28	160.31 ± 149.50	0.504	0.615
ALB (g/L)	30.18 ± 6.58	30.02 ± 5.20	−0.177	0.860
PCT (ng/L)	16.28 ± 31.52	14.20 ± 25.52	−0.470	0.639
Na^+^ (mmol/L)	139.24 ± 5.93	138.70 ± 6.09	−0.537	0.592
K^+^ (mmol/L)	3.93 ± 0.80	3.86 ± 0.51	−0.789	0.431
Glu (mmol/L)	9.33 ± 3.01	9.29 ± 2.67	−0.103	0.918
Lac (mmol/L)	2.76 ± 3.05	2.21 ± 2.15	−1.428	0.155
BMI (kg/m^2^)	22.15 ± 3.28	22.69 ± 3.19	1.015	0.311
APACHEII	15.04 ± 5.99	14.15 ± 7.15	−0.777	0.438
GCS	11.51 ± 3.86	9.63 ± 3.90	2.941	0.004

**Table 4:** Independent predictors of delirium in ICU derived from multivariate regression analysis performed on the derivation cohort

Risk factor	β coefficient	SE	Wald value	OR	RR (95% CI)	P	Points in weighted scoring mode
History of hypertension	1.474	0.636	5.374	4.367	1.256–15.188	0.02	3
Hypoxaemia	1.219	0.513	5.642	3.382	1.237–9.245	0.018	2
Use of benzodiazepines	1.505	0.605	6.181	4.503	1.375–14.746	0.013	3
Deep sedation	1.206	0.610	3.909	3.339	1.011–11.035	0.048	2
Mechanical ventilation	1.266	0.043	4.338	3.547	1.077–11.679	0.037	2
Sepsis	1.247	0.471	7.020	3.480	1.383–8.745	0.008	2

### Validation of prediction model

We performed a univariate analysis using 16 variables from Table 1 to identify factors associated with ICU delirium: history of hypertension, history of diabetes, hypoxaemia, hypotension, deep sedation, benzodiazepines, metabolic acidosis, sepsis (Table 2).

Multivariate regression analysis demonstrated that history of hypertension, hypoxaemia, use of benzodiazepines, deep sedation, need for mechanical ventilation and infection were independent risk factors for delirium in critically ill patients (Table 4). The developed logistic regression model was based on these 6 independent predictors: logit(P) = − 3.563 + 1.474 * for history of hypertension + 1.219 * for hypoxaemia + 1.505 * for use of benzodiazepines + 1.206 * for deep sedation + 1.266 * for need for mechanical ventilation + 1.247 * for sepsis. The closer P was to one, the greater the likelihood of delirium, and the closer P was to zero, the smaller the likelihood of delirium.

**Fig. 2:**
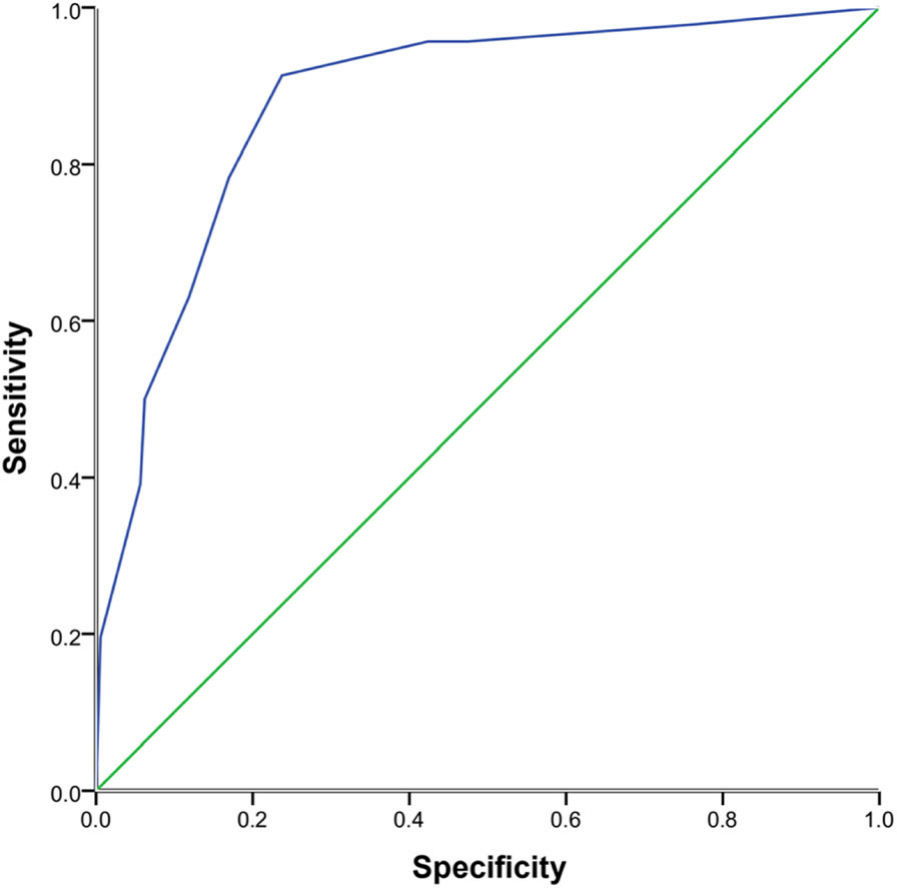
Area under the receiver operating characteristic curve (AUROC) of the derivation cohort

The six risk factors of the β coefficient were divided by the smallest β coefficient in Table 5, multiplied by 2, and rounded to the nearest integer to obtain the weighted score of each predictor. The AUROC of delirium in the ICU was 0.862 ± 0.03 [*P* < 0.001, 95% CI (0.803–0.921)], which were determined in the risk score of the derivation cohort, (Fig. 2).

**Table 5:** Hosmer-Lemeshow test used for the predicted and observed cases of delirium in the ICU in the validation cohort

ICU delirium risk score	Total cases	Observed cases	Predicted cases
0 ~ 4	34	3	1.68
5 ~ 9	40	12	12
10 ~ 14	7	3	3.7

**Fig. 3:**
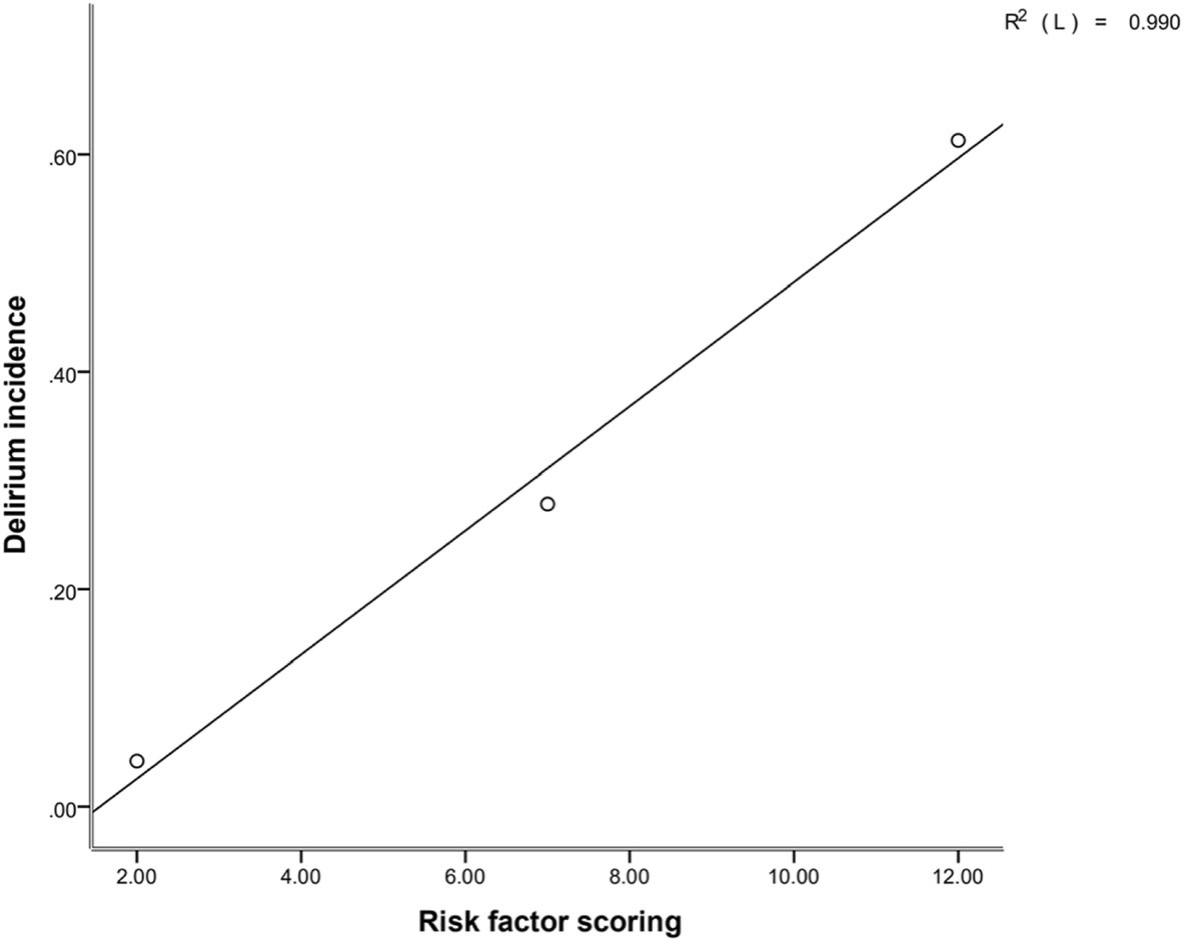
The connection between risk factors and delirium incidence in ICU patients

**Fig. 4:**
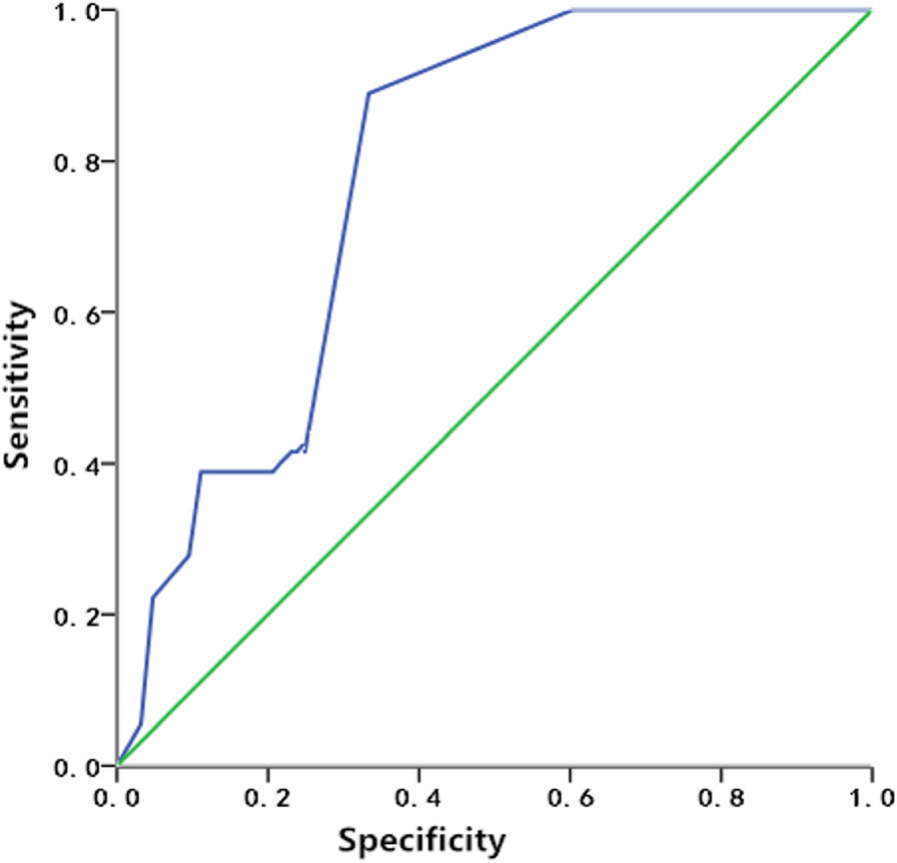
Area under the receiver operating characteristic curve (AUROC) of the validation

The associations of risk factors with the ICU delirium incidence in the derivation cohort were positive such that the higher the risk score, the higher the incidence. The occurrence of ICU delirium was 4.42% for a score of 0–4, 27.85% for a score of 5–9 and 61.29% for a score of 10–14 (Fig. 3). The formula for the association was as follows: expected incidence of delirium = − 0.089 + 0.059 * risk score, which was calculated as the risk score and the expected incidence of delirium in ICU patients in the validation cohort. The AUROC was 0.739 ± 0.06 [*P* = 0.002, 95% CI (0.620 ~ 0.857)] in the validation cohort (Fig. 4).

According to the predictive model, a Hosmer-Lemeshow test was used to confirm the formula for the risk score. The findings confirmed that there were no significant differences between the predicted and observed cases of delirium in the validation cohort (χ2 = 0.000, *P* > 0.05) (Table 5).

## Discussion

We found a 21.1% incidence of delirium in critically ill patients, which is consistent with Von Rueden’s finding [15] of a 24% incidence of delirium after trauma but is lower than the rate after liver transplantation reported by Beckmann [16]. The APACHE II score system is used to evaluate the severity and prognosis of critically ill patients. Our study showed that the average APACHE II score was 15, which indicated a relatively mild state and lower incidence than the score of 24 reported by Pandharipande [17] and the score of 29 reported by Peterson [18]. In addition, another reason for the lower incidence of delirium in our study was our heterogenous population, which included surgical and medical patients. The reason for the high prevalence of delirium remains poorly understood; however, recent studies have suggested that cognitive disorders and simultaneously slow brain wave activity are caused by extensive brain oxidative disturbances. Numerous studies have demonstrated that the occurrence of delirium increased significantly when patients had severe sepsis [19, 20]. Our review also showed that sepsis was independently associated with the development of delirium. Nerve neurons are particularly vulnerable to hypoxia and are susceptible to hypoxic encephalopathy. In turn, Hypoxia may further destroy nerve cells, causing repeated or acute changes in mental states. Therefore, oxygenation plays a vital role in critically ill patients, and consequently, a balance between oxygen supply and demand may reduce the incidence of delirium [18]. In addition, hypertension is often associated with neurological damage, such as memory decline and cognitive impairment [21]. Chronic hypertension could result in white matter damage and may contribute to dementia [22]. Therefore, patients in the ICU with hypertension may have increased risks of hypoxia, hypoperfusion of neurons, and than increasing the occurence of delirium.

In accordance with previous studies [23, 24], the use of benzodiazepine was reported to be an independent risk factor for the development of delirium in critically ill patients. Benzodiazepine can block the γ-aminobutyric acid pump, affecting reuptake, which thus results in increased levels of the inhibitory neurotransmitter [25]. A meta-analysis of 1235 candidate predictors [26] showed that non-benzodiazepine sedation was associated with shorter hospitalization (decreased by 1.6 days), reduced mechanical ventilation (decreased by 1.9 days) and lower mortality than benzodiazepine sedation. Therefore, non-benzodiazepine sedatives, such as propofol and dexmedetomidine, are preferred for intensive care patients. Moreover, some results indicated that the generation level of sedation may influence the incidence of delirium [27]. Kollef, M.H [28]. and Treggiari, M [29]. also demonstrated that compared with light sedation, deep sedation increased the number of ventilation days, length of ICU stay and occurrence of post-ICU syndrome. Interestingly, other studies revealed no obvious mental disorders with daily interruptions of sedation programmes [30]. The discomfort and communication barriers caused by tracheal intubation, alarm sounds from the machine, confined limb movements and sleep wake cycle disorders can all cause these patients to be prone to delirium [31].

Our model reliably predicted the development of delirium for the entire ICU length of stay on the basis of 6 risk factors readily available within 24 h of ICU admission. These findings confirm that the model has additional value in daily practice. Several limitations of this study need to be addressed. First, in this performance study, the CAM-ICU was measured at two time points on one day. Considering the fluctuating nature of delirium, all patients were screened more often if needed. Second, delirium was classified into subtypes of hypoactive, hyperactive, and mixed delirium [32]. The incidence of delirium was lower in this study, which may be because hypoactive delirium is not easily identified by medical personnel. This finding suggested that future clinical work should aim to further strengthen training programmes for attending nurses and physicians. Third, disease type and environmental factors were not defined as risk factors. Finally, one study showed that C-reactive protein (CRP) measured on ICU stay was associated with the development of delirium [33], as many aetiological factors associated with delirium will produce inflammation, which elevates CRP levels. Accordingly, we did not include CRP in the model.

In conclusion, we can now easily identify patients who have a high risk of developing delirium following ICU admission using only six predictors. This will facilitate targeted initiation of preventive measures. Therefore, the model should be used daily in intensive care practice.

## Data Availability

The datasets used and/or analysed during the current study are available from the corresponding author on reasonable request.
